# The role of Lipoxin A4 in Cystic Fibrosis Lung Disease

**DOI:** 10.5936/csbj.201303018

**Published:** 2013-12-06

**Authors:** Valérie Urbach, Gerard Higgins, Paul Buchanan, Fiona Ringholz

**Affiliations:** a National Children's Research Centre, Crumlin, Dublin 12, Ireland; b Institut National de la Santé et de la Recherche Médicale, U845, Faculté de Médecine Paris Descartes - Site Necker - 156 rue Vaugirard 75015, Paris, France

## Abstract

In Cystic Fibrosis (CF), mutations of the CFTR gene result in defective Cl^−^ secretion and Na^+^ hyperabsorption by epithelia which leads to airway lumen dehydration and mucus plugging and favours chronic bacterial colonization, persistent inflammation and progressive lung destruction. Beyond this general description, the pathogenesis of CF lung disease remains obscure due to an incomplete understanding of normal innate airway defense. This mini-review aims to highlight the role of the pro-resolution lipid mediator, Lipoxin A_4_, which is inadequately produced in CF, on several aspects of innate immunity that are altered in CF airway disease.

## Cystic Fibrosis airway disease

Cystic Fibrosis (CF) is a lethal genetic disorder which results from a mutation of the gene coding for the Cystic Fibrosis Transmembrane conductance Regulator (CFTR), a cyclic AMP-dependent Cl^-^ channel [1]. Cystic Fibrosis affects various organs in which the CFTR protein is normally expressed. The major clinical features of CF are chronic pulmonary disease, exocrine pancreatic insufficiency and male infertility, however, the lung disease is the main cause of morbidity and mortality in CF [2–4]. Healthy airways are lined by an epithelial layer that plays a major role in defense against inhaled pathogens involving several specialized epithelial functions including; mechanical barrier, adequate surface hydration due to an intricate regulation of ions and water transport, mucus secretion, production of antimicrobial peptides, expression of receptors that recognize pathogen associated molecular patterns (PAMPs), secretion of cytokines that control the local immune responses in the airway lumen. In CF mutations of the CFTR gene results in defective Cl^-^ secretion and Na^+^ hyperabsorption by airway epithelia [5, 6]. This contributes to reduction of the periciliary fluid volume, the airway lumen dehydration, reduction of the periciliary fluid volume and mucus plugging [7]. This results in an impaired mucociliary clearance of pathogens from the lung, favouring chronic bacterial colonization, persistent inflammation and progressive destruction of the lung [8]. In addition to the abnormality of epithelial ion transport, other epithelial dysfunctions have been described in chronically inflamed and infected CF airways, intrinsic pro-inflammatory properties, amplified inflammatory responses to infections and reduced bacterial clearance. However, beyond this general description, the pathogenesis of the CF lung disease remains obscure.

## Anti-inflammatory therapy in Cystic Fibrosis

Whilst the field continues to celebrate the success, for a minority of people with CF, in achieving therapeutic benefits via CFTR modulation strategies, reduction of lung inflammation and restoration of airway hydration / muco-ciliary clearance remain core goals of CF therapy for the majority. Several anti-inflammatory approaches have been examined in CF, however, the ideal anti-inflammatory drug is not yet available [9]. A recent systematic review of the risks and benefits of Inhaled corticosteroids (ICS) in CF, examining evidence from 13 trials, concluded that there is insufficient evidence to establish whether ICS are beneficial in CF, but withdrawal in those already taking them has been shown to be safe [10]. It is established that ICS use can have adverse effects on growth. A systematic review of the efficacy of non-steroidal anti-inflammatory drugs in CF concluded that treatment with high-dose ibuprofen was associated with a significantly lower annual rate of decline in lung function (especially in children), however, the adoption of ibuprofen into therapy has not been universally accepted [11, 12]. Redressing the imbalance in fatty acid metabolism described in CF, by supplementation of Docosahexaenoic Acid may be helpful, and efforts are ongoing to evaluate the potential therapeutic benefit [13].

## The Specialized Pro-resolving Mediators

New perspectives have emerged in inflammation research with the discovery of new classes of biologically active lipid mediators playing specialised roles in the active resolution of inflammation – the “specialized pro-resolving mediators” (*SPM*) [14]. Furthermore, the acute inflammatory response is a protective mechanism that evolved to eliminate invading organisms and yet be self-limited with an active resolution phase designed to restore tissue homeostasis. The resolution phase is carried out by the actions of *SPM* which are non-immunosuppressive [14]. The temporal evolution of acute inflammation toward its active resolution is directed by the sequential expression and activity of characteristic classes of eicosanoid mediator in a process termed “class switching” [15]. Prostaglandins are biosynthesised early, initiating the inflammatory response. Leukotrienes follow, typified by Leukotriene B_4_ (LTB_4_) which plays its role in amplification and propagation of inflammation [15] acting in concert with the cytokine Interleukin 8 (IL-8) as a potent neutrophil chemo-attractant [16, 17]. Lipoxin A_4_ (LXA_4_) is the first eicosanoid of the *SPM* family to be expressed in the active resolution phase of inflammation. LXA_4_ production is followed by the biosynthesis of Resolvins and Protectins at the inflammatory site. These *SPM* are biosynthesized *in vivo* in inflammatory exudates from essential fatty acids; Lipoxins (LX) from arachidonic acid; E-series resolvins (Rv) from Omega-3 Eicosapentaenoic acid (EPA); D-series resolvins and protectins (PD) from Docosahexaenoic acid (DHA) [14].

## Anti-inflammatory properties of Lipoxin A_4_

The anti-inflammatory properties of LXA_4_ have been reported in a wide variety of tissues. LXA_4_ inhibits nuclear factor-kappaB activation, which results in inhibition of pro-inflammatory cytokine release and inhibition of inflammatory responses in microglial cells, astrocytoma cells, macrophages, peripheral blood mononuclear cells (PBMC), polymorphonuclear leukocytes (PMN) and intestinal epithelial cells [18–20]. LXA_4_ inhibits neutrophil functions, most notably inhibiting LTB_4_ induced neutrophil chemotaxis, neutrophil adherence and transmigration across intestinal epithelium and endothelium and inhibiting superoxide anion and peroxynitrite generation [14, 21–23]. LXA_4_ facilitate neutrophil apoptosis [24] and stimulates phagocytosis of apoptotic neutrophils by macrophages [25, 26]. This is a critical point since delayed neutrophil apoptosis appears to be a component of the pathophysiology in patients with inflammatory diseases, including cystic fibrosis [27, 28] and frequently correlates with disease severity and outcome. In the airways, *in vitro* and *in vivo* studies also report that LXA_4_ displays diverse and potent anti-inflammatory actions [19, 29, 30]. In human airways, LXA_4_ suppresses IL-8 production by leukocytes and bronchial epithelial cells [30, 31]. LXA_4_ was shown to arrest neutrophilic inflammation and decrease infection in a mouse model of chronic airway inflammation and infection [32]. LXA_4_ has been proposed as a novel regulators of adaptive immunity and may have therapeutic potential in chronic immune disorders [33]. The pro-resolution properties of LXA_4_ are mediated by the ALX/FPR2 receptor. The ALX/FPR2 is a G protein-coupled receptor of seven-transmembrane domains that is expressed mainly by mammalian phagocytic leukocytes. The known effect of ALX/FPR2 receptor in the in host defense and inflammation is not only triggered by LXA_4_ since other pro-resolution mediators such as resolving D1 also mediate their effects through this receptor [34, 35].

## Abnormal eicosanoid class switching in Cystic Fibrosis

LXA_4_ is produced by lipoxygenase (LO) interactions resulting from trans-cellular cooperations [36] of neutrophils [37], eosinophils [38], alveolar macrophages [39], platelets [40] or airway epithelial cells [41], each expressing different Lipoxygenase (LO) enzymes which act in sequence in LXA_4_ biosynthesis [16, 42]. The 5-LO expressed by neutrophils can utilise the 15(S)-hydroxyeicosatetranoic acid released by epithelial cells as substrate to synthesize lipoxins [37]. The platelet 12-LO [40], the macrophages or epithelial 15-LO [39, 19] are each able to transform Leukotriene A_4_, released by neutrophils, into LXA_4_. Up-regulation of 15-LO activity generates an inverse relationship between LTB_4_ and LXA_4_ biosynthesis. The activity of 15-LO promotes LXA_4_ biosynthesis and blocks leukotriene biosynthesis, both as a result of 15-LO products competing for flux at the 5-LO level, and by diversion of the intermediate Leukotriene A_4_ away from LTB_4_ towards LXA_4_ biosynthesis [15, 41, 42]. The levels of LXA_4_ have been reported to be decreased in chronic airway inflammatory disease such as asthma, chronic obstructive pulmonary disease and CF [29, 32, 43, 44]. A decreased proportion of pro-resolving compounds (LXA_4_) compared to pro-inflammatory (LTB_4_) is associated with decrease of lung function [45]. The absolute content of LXA_4_ concentration in CF BAL fluid from patients with CF is not significantly different from controls [46]. However, a significant suppression in LXA_4_/neutrophil ratios in BAL fluid of patients with CF compared with pulmonary inflammatory controls was reported [32, 46]. More specifically, *in vitro* studies support a role for CFTR in LXA_4_ production. CFTR inhibition reduced LXA_4_ synthesis by 50% during platelets/ PMN co-incubation by inhibiting the lipoxin synthase activity of platelets 12-LO. Platelets from patients with CF generated 40% less LXA_4_ compared to healthy subject [47]. The decreased LXA_4_ production in CF provides a mechanistic explanation of the failure to actively resolve acute airway inflammation seen in these patients.

## Lipoxin A_4_ regulates ion transport and the airway surface liquid height

**Figure 1:**
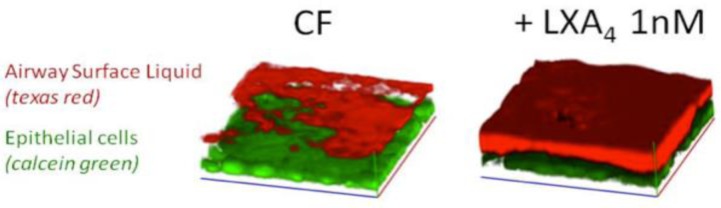
LXA_4_ restores the Airway Surface Liquid (ASL) layer in CF bronchial epithelium. Live cell imaging using confocal microscopy of bronchial epithelium in primary culture from a child with CF before and after treatment with LXA_4_. The bronchial epithelial cells are stained in green using *calcein green* and the ASL in red using dextran coupled to *texas-red*.

LXA_4_ stimulates a rapid and transient intracellular Ca^2 +^ increase and induces Cl^−^ secretion through human bronchial epithelial cells by Ca^2 +^-activated Cl^−^ channel and not CFTR [48]. Furthermore, LXA_4_ effects on ion transport lead into an increase of the airway surface liquid (ASL) layer height in models of fully differentiated bronchial epithelia derived from primary culture of bronchial brushings from patients with CF (Figure 1). LXA_4_ exerts this effect on the ASL dynamics via the ALX/FPR2 receptor which is expressed in the apical membrane of airway epithelial cells. The sustained increase in ASL height induced by LXA_4_ in non-CF and CF bronchial epithelial results from stimulation of an intracellular calcium signal and Ca^2 +^-activated Cl^−^ secretion via NPPB sensitive Cl^−^ channels [49]. LXA_4_ thus restores Cl^−^ secretion and adequate ASL height which are affected in CF airways, highlighting a role for LXA_4_ in the control of innate immune defence. The inadequate endogenous LXA_4_ biosynthesis in CF contributes to the reduce ASL volume and impaired mucociliary clearance in addition to alter resolution of inflammation in the airway, thus amplifying the vicious circle of airway dehydration, chronic infection and inflammation.

## Lipoxin A_4_ regulates airway epithelial integrity

Epithelial repair is a key process required to maintain epithelial barrier integrity and respiratory function, however in CF, repeated infections and inflammatory insults result in damage to the airways, triggering the repair process [50]. Epithelial repair initially involves cell spreading and migration, followed by proliferation to repopulate the denuded area that has been created by injury [51–53]. This is then followed by differentiation of the epithelium [54]. Recent research suggests that epithelial repair and differentiation of the CF airway epithelium is down-regulated or delayed [55–59]. More specifically, cell migration and proliferation appear to both be reduced during repair in CF bronchial epithelial cells compared to non-CF [60]. This delay in repair of the CF epithelium renders the lung more susceptible to ongoing bacterial infection and thus may lead to more epithelial damage [61]. It was recently reported that LXA_4_ can trigger epithelial cell migration and proliferation and thus play a role in repair of corneal and bronchial epithelia [62–64, 65–67]. LXA_4_ triggers an increase in migration, proliferation, and wound repair of non-CF and CF bronchial epithelia. These responses to LXA_4_ are mediated by the ALX/FPR2 receptor via the downstream activation of K_ATP_ channels and ERK MAP kinase phosphorylation [60]. This effect of LXA_4_ both ion transport and repair are consistent with the role of ion channels in two key processes of repair, migration and proliferation [68]. In particular potassium channels have been shown in numerous cell types to be involved in cell migration and proliferation [57, 58, 69–72]. Furthermore, LXA_4_ enhances airway epithelial tight junction formation. LXA_4_ stimulates ZO-1, claudin-1 and occludin expression and trafficking at the apical membrane resulting in enhanced transepithelial electrical resistance in human airway epithelia [73]. Therefore the reduced levels of LXA_4_ in the CF airways [74] may account for the reduced capacity for epithelial repair in the CF epithelium.

## Therapeutic potential for LXA_4_ in cystic fibrosis treatment

Clearance of airway secretions has been a first line therapy for patients with CF and a variety of airway clearance therapies have been developed [75, 76]. One of the greatest challenges into reversing the CF defect in the airways is to design strategies to overcome the absence of functional CFTR by stimulating chloride secretion via alternative pathways, thus restoring airway hydration and mucociliary clearance. This can be achieved via the activity of calcium-activated chloride channels stimulated by agents that raise the intracellular calcium concentration. This strategy has been plagued by the attendant side effects of the amplification of the calcium dependant pro-inflammatory response resulting in the undesirable activation of NFκB [26]. Thus identification of agents, particularly natural endogenous biologicals, that stimulate alternative non-CFTR Cl^−^ secretory pathways and promote ASL hydration and optimal ASL height recovery are likely to be of therapeutic benefit in improving mucociliary clearance in patients with CF. The effects of LXA_4_ inhalation has been evaluated in a pilot study of eight asthmatic and healthy adult subjects. The challenge was tolerated, had no adverse effect on pulse or blood pressure and demonstrated favourable effects on specific airway conductance [77].

## Conclusion and future prospect

**Figure 2:**
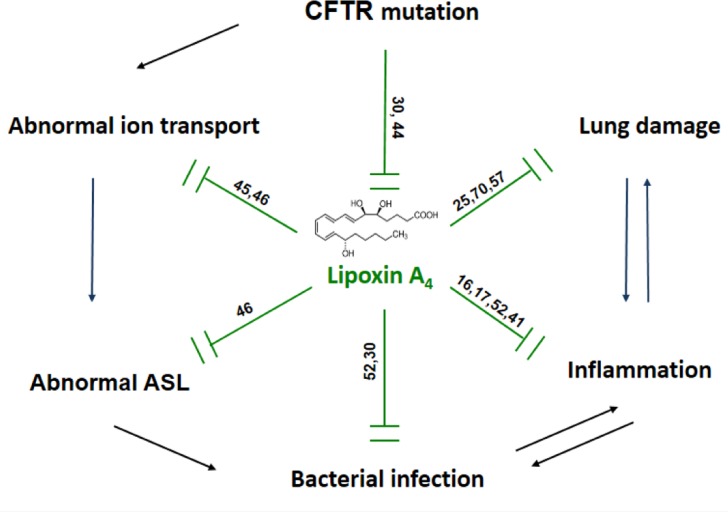
Pleiotropic effects of LXA_4_. LXA_4_ which is abnormally produced in Cystic Fibrosis controls various airway physiological functions. LXA_4_ regulates bronchial epithelium ion transport, enhances the airway surface liquid (ASL) height, protects epithelial barrier integrity and reduces inflammation.

Taken together, the discovery of the multiple LXA_4_ functions in restoring bronchial epithelium ion transport in enhancing ASL height, in restoring epithelial barrier function and in reducing inflammation might provide significant advance in improving quality of life and longevity for CF patients (Figure 2).
